# Early life serum neurofilament dynamics predict neurodevelopmental outcome of preterm infants

**DOI:** 10.1007/s00415-021-10429-5

**Published:** 2021-02-10

**Authors:** Katharina Goeral, Annalisa Hauck, Andrew Atkinson, Michael B. Wagner, Birgit Pimpel, Renate Fuiko, Katrin Klebermass-Schrehof, David Leppert, Jens Kuhle, Angelika Berger, Monika Olischar, Sven Wellmann

**Affiliations:** 1 Division of Neonatology, Department of Pediatrics and Adolescent Medicine, Pediatric Intensive Care and Neuropediatrics, Comprehensive Center for Pediatrics, Medical University Vienna, Vienna, Austria; 2 Division of Neonatology, University Children’s Hospital Basel (UKBB), University of Basel, Basel, Switzerland; 3 Division of Pediatric Pharmacology and Pharmacometrics, University Children’s Hospital Basel (UKBB), Basel, Switzerland; 4 Neurologic Clinic and Policlinic, Departments of Medicine, Biomedicine and Clinical Research, University Hospital Basel, University of Basel, Basel, Switzerland; 5 Division of Neonatology, Campus Hospital St. Hedwig, University Children’s Hospital Regensburg (KUNO), University of Regensburg, Steinmetzstr 1-3, 93049 Regensburg, Germany

**Keywords:** Neurofilament, Brain damage, Neurodevelopment, Children, Biomarker

## Abstract

**Background and purpose:**

To determine whether neurofilament light chain (NfL), a promising serum and cerebrospinal fluid (CSF) biomarker of neuroaxonal damage, predicts functional outcome in preterm infants with neonatal brain injury.

**Methods:**

Our prospective observational study used a sensitive single-molecule array assay to measure serum and CSF NfL concentrations in preterm infants with moderate to severe peri/intraventricular hemorrhage (PIVH). We determined temporal serum and CSF NfL profiles from the initial diagnosis of PIVH until term-equivalent age and their association with clinical and neurodevelopmental outcome until 2 years of age assessed by Bayley Scales of Infant Development (3rd edition). We fitted univariate and multivariate logistic regression models to determine risk factors for poor motor and cognitive development.

**Results:**

The study included 48 infants born at < 32 weeks of gestation. Median serum NfL (sNfL) at PIVH diagnosis was 251 pg/mL [interquartile range (IQR) 139–379], decreasing markedly until term-equivalent age to 15.7 pg/mL (IQR 11.1–33.5). CSF NfL was on average 113-fold higher (IQR 40–211) than corresponding sNfL values. Additional cerebral infarction (*n* = 25)-but not post-hemorrhagic hydrocephalus requiring external ventricular drainage (*n* = 29) nor any other impairment-was independently associated with sNfL. Multivariate logistic regression models identified sNfL as an independent predictor of poor motor outcome or death at 1 and 2 years.

**Conclusions:**

Serum neurofilament light chain dynamics in the first weeks of life predict motor outcome in preterm infants with PIVH.

**Supplementary Information:**

The online version contains supplementary material available at 10.1007/s00415-021-10429-5.

## Introduction

One in ten infants are born preterm worldwide [1]. Despite steadily increasing survival rates, long-term neurodevelopmental outcome remains worrying, due mainly to brain damage, specifically peri/intraventricular hemorrhage (PIVH) [2,3], diagnosed in the first few days of life by cerebral ultrasound (cUS) and graded using the classification by Papile et al. [4] revisited by Volpe [5]. The anatomic and neurobiological substrates of the neurologic deficit relate to PIVH grade and involve a combination of primary destructive effects, e.g., brain tissue infarction, and secondary developmental disturbances such as altered CSF circulation, elevated intracranial pressure and impaired myelin development [6].

Neuronal scaffolding comprises neurofilaments (Nf) made of four highly specific protein subunits: the Nf triplet [light chain (NfL), medium and heavy chain] and α-internexin (in the central nervous system) or peripherin (in the peripheral nervous system). Acute or chronic neuronal damage releases Nf fragments into the CSF and eventually the blood compartment [7]. Highly sensitive single-molecule array (Simoa) immunoassays have improved NfL detection, particularly in peripheral blood, creating a promising and readily accessible biomarker for neuroaxonal injury, including in slowly progressive or even presymptomatic disease such as Alzheimer’s [8].

Our group recently found elevated plasma NfL in preterm newborns with PIVH [9], prompting the hypothesis that peripheral NfL is related to PIVH severity and neurodevelopmental outcome between 1 and 2 years of age.

## Methods

We conducted the prospective observational study at the Department of Pediatrics and Adolescent Medicine, Medical University Vienna, Austria, between May 2011 (first patient in) and June 2020 (last patient completing 2-year follow-up) following approval by the competent ethics committee (EK 252/2011) and written informed consent from the parents.

We included preterm infants born at < 32 weeks of gestation with evidence of severe (grade 3–4) PIVH on cUS screening in the first days of life. We also included infants with grade 2 PIVH and evidence of early post-hemorrhagic hydrocephalus (PHH). Non-inclusion criteria were chromosomal aberration, major malformation requiring surgery in the first month of life, a priori palliative care, death in the first 5 days of life, and absence of parental consent. Infants with severe PHH received extraventricular drainage (EVD) to remove CSF when ventricular dilatation exceeded 4 mm above the 97th centile [10]. If ventricular expansion continued, a permanent ventriculo-peritoneal shunt was implanted for outpatient care.

Peri/intraventricular hemorrhage was graded using the amended Papile et al. classification: [4,5] grade 1 (ineligible for our study) denotes hemorrhage originating in the germinal matrix and remaining confined to this highly vascularized subventricular region; grades 2 or 3 refer to the volume and extent of hemorrhage into the ventricular system. Infarction, equivalent to grade 4 PIVH, may occur together with or separately from blood in the ventricular system [5].

The study comprised three visits for all infants with or without CSF drainage: PIVH visit (initial cUS diagnosis of PIVH), PIVH-plus visit (cUS confirmation of PIVH), and a final term-equivalent age (TEA) visit. Infants requiring CSF drainage for severe PHH received the following additional visits: EVD visit (first CSF removal), EVD-plus visit (subsequent CSF removal) and in some cases additional visits (CSF 3 or 4) at subsequent removals.

The primary endpoint was neurodevelopment at 2 years of age. Cognitive and motor development was examined in an assessment consisting of clinical examination including anthropometric measurement, structured neurologic assessment, and developmental assessment using the Bayley Scales of Infant and Toddler Development (3rd edition; Bayley-III) [11]. A secondary endpoint was neurodevelopment at 1 year of age, also assessed using Bayley-III. Assessors were blinded for NfL findings.

Serum samples were collected and processed according to a standard operating procedure involving transfer to a central laboratory, centrifugation, preparation of aliquots, and storage at − 80 °C until batch-wise analysis. No sample had previously been thawed. Technicians were blinded to clinical information and outcome. NfL was assayed by Simoa as previously described; [12] intra and interassay variabilities were < 10%. The few samples with intra-assay coefficients of variation > 20% underwent repeated measurement.

In terms of descriptive statistics, we tested for differences in neurodevelopmental outcome between infants with poor outcome or death and those with better outcome between 1 and 2 years using Student’s *t* test for normally distributed continuous variables, the Mann–Whitney-Wilcoxon test for non-normally distributed continuous variables, and Pearson’s *χ*^2^ test for dichotomous variables.

Univariate and multivariate-adjusted logistic models were fitted with neurodevelopmental outcome as the dependent variable and the baseline characteristics listed in Table 1 as independent variables. The predictive power of the model was validated using cross-validation. Forest plots were used to compare areas under the curve (AUC) from the resulting plots. Receiver operating characteristic curves were drawn from univariate models [13]. To investigate the development of sNfL over time in more detail, an adjusted linear mixed effects model was fitted with measured sNfL as the dependent variable and the variables listed in Table 1 as fixed-effect regressors with random intercept and slope effects per patient. In a further step to compare sNfL trajectories between the two neurodevelopmental outcome groups, we fitted a general additive model with sNfL as the dependent variable and used restricted cubic splines to model potential non-linear time effects.

**Table 1:** Baseline and follow-up characteristics stratified by 2-year endpoints; motor skills (left) and cognitive skills (right)

Characteristics	2-year motor skills (2 missing values)			2-year cognitive skills (2 missing values)		
	Better skills	Poor skills or death	*p*	Better skills	Poor skills or death	*p*
*N*	22	24	–	23	23	–
Died (*N*/%)	0 (0)	8 (33.3)	**0.01**	0 (0)	8 (34.8)	**0.006**
Mother’s age (years) [M (IQR)]	31 [27, 35]	31 [28, 34]	0.9	31 [28, 34]	31 [28, 34]	0.9
Preeclampsia (*N*/%)	2 (9.1)	0 (0.0)	0.4	2 (8.7)	0 (0.0)	0.5
AIS (*N*/%)	10 (45.5)	15 (62.5)	0.4	11 (47.8)	7 (30.4)	0.5
Antenatal steroids, full course (*N*/%)	4 (18.2)	3 (12.5)	0.9	3 (13.0)	4 (17.4)	0.9
Cesarean delivery (*N*/%)	17 (77.3)	16 (66.7)	0.9	17 (73.0)	16 (69.6)	0.9
Sex (female) (*N*/%)	9 (40.9)	4 (15.7)	0.1	9 (39.1)	4 (17.4)	0.2
GA (weeks) [M (IQR)]	26.7 [24.7, 28.3]	25.5 [24.4, 26.6]	0.2	26.6 [24.6, 28.3]	25.6 [24.6, 26.6]	0.4
Birth weight (g) [M (IQR)]	926 [630, 1308]	810 [695, 931]	0.3	870 [588, 1226]	824 [738, 933]	0.8
Apgar 5 min < 6 points (*N*/%)	2 (9.1)	3 (12.5)	0.6	2 (8.7)	3 (13.0)	0.6
Neonatal infection (*N*/%)	21 (95.5)	22 (91.7)	0.9	22 (95.7)	21 (91.3)	0.9
PDA medical treated (*N*/%)	9 (40.9)	10 (41.7)	0.9	10 (43.5)	9 (39.1)	0.7
PDA surgery (*N*/%)	3 (13.6)	3 (12.5)	0.9	2 (8.7)	4 (17.4)	0.7
Postnatal steroids (*N*/%)	4 (18.2)	3 (12.5)	0.9	3 (13.0)	4 (17.4)	0.9
ROP grade > 1 (*N*/%)	11 (50.0)	12 (50.0)	0.9	12 (52.2)	11 (47.8)	0.9
BPD moderate or severe (*N*/%)	8 (36.4)	4 (16.7)	0.2	8 (34.8)	4 (17.4)	0.3
NEC grade > 1 (*N*/%)	4 (18.2)	7 (29.2)	0.6	6 (26.1)	5 (21.7)	0.9
PIVH average [M (IQR)]	3.0 [2.5, 3.0]	3.3 [3.0, 3.5]	**0.001**	3.0 [2.5, 3.0]	3.3 [3.0, 3.5]	**0.02**
Infarction (*N*/%)	6 (27.3)	16 (66.7)	**0.02**	7 (30.4)	15 (65.2)	**0.04**
PHH with CSF drainage (*N*/%)	11 (50.0)	17 (70.8)	0.3	12 (52.2)	16 (69.6)	0.4
Shunt (*N*/%)	6 (27.3)	9 (37.5)	0.7	6 (26.1)	9 (39.1)	0.5
Shunt infection (*N*/%)	2 (9.1)	3 (12.5)	0.9	3 (13.0)	2 (8.7)	0.9
Higher SES (*N*/%)	12 (54.5)	9 (37.5)	**0.05**	12 (52.2)	9 (39.1)	**0.03**
Early log_10_ sNfL [M (IQR)]	2.4 [2.1, 2.5]	2.6 [2.5, 2.7]	**0.01**	2.4 [2.2, 2.6]	2.6 [2.3, 2.7]	0.3
Highest log_10_ sNfL [M (IQR)]	2.4 [2.1, 2.5]	2.7 [2.6, 2.9]	**0.001**	2.5 [2.2, 2.7]	2.6 [2.5, 2.8]	0.2

*AIS* amnion infection syndrome, *GA* gestational age, *PDA* patent ductus arteriosus, *ROP* retinopathy of prematurity, *BPD* bronchopulmonary dysplasia, *NEC* necrotizing enterocolitis, *PIVH* peri/intraventricular hemorrhage, average between PIVH grade left and right side, *PHH* post-hemorrhagic hydrocephalus, *SES* socio-economic standard, *M* median, *CSF* cerebrospinal fluid

## Results

**Fig. 1:**
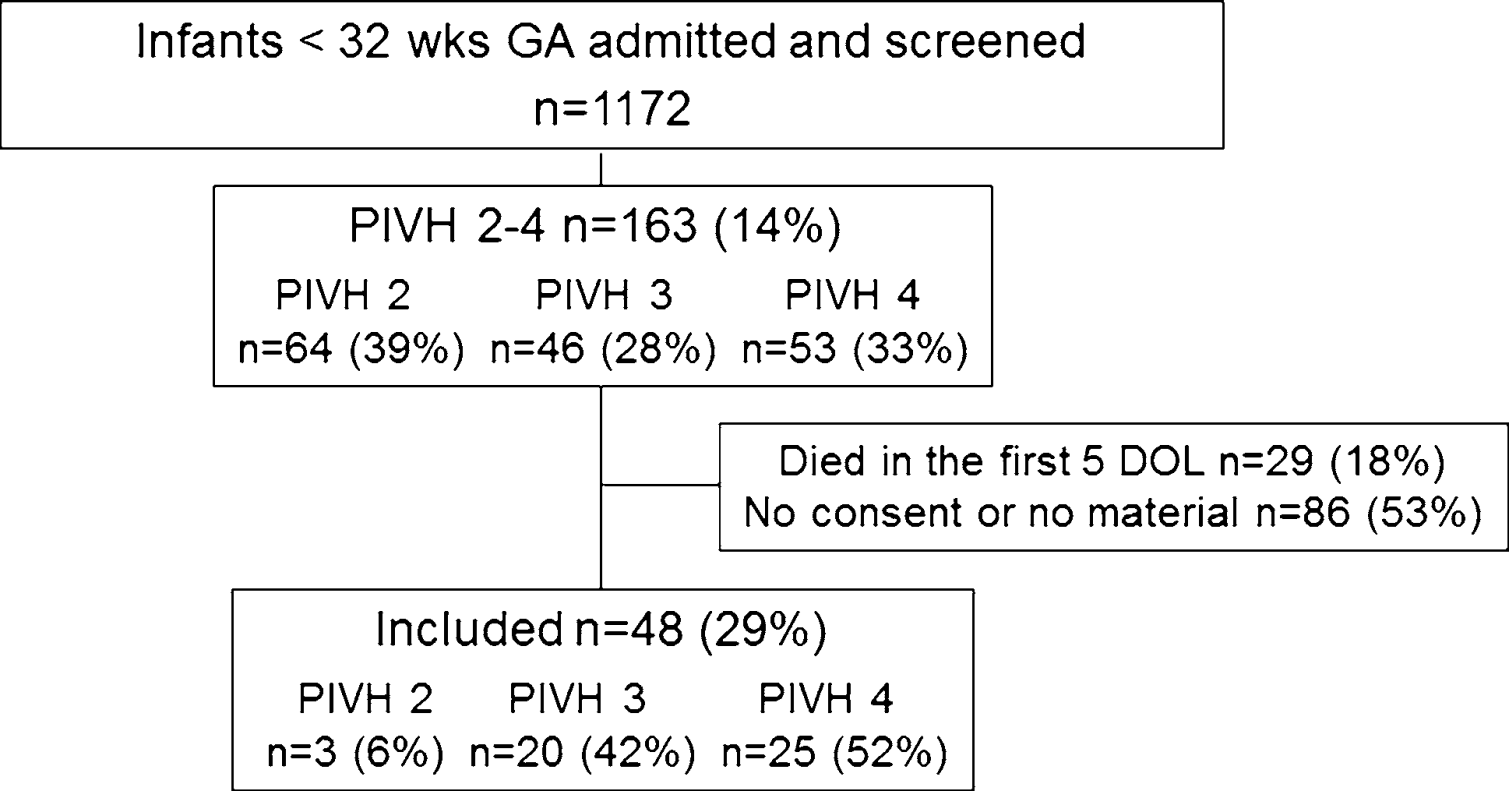
CONSORT flow chart; *PIVH* peri/intraventricular hemorrhage, *GA* gestational age at birth, *DOL* day of life

Of the total 48 infants (Fig. 1, CONSORT flow diagram) infarction was detected in 25 (52%): in both hemispheres (*n* = 5), with contralateral PIVH 3 (*n* = 12), with contralateral PIVH 2 (*n* = 7), and no contralateral hemorrhage (*n* = 1). In 20 infants (42%) the highest grade detected was PIVH 3: bilateral (*n* = 13), with contralateral PIVH 2 (*n* = 5), and no contralateral hemorrhage (*n* = 2). In three infants (6%) PIVH 2 was the highest grade detected, bilateral in each case. PHH was present in 33 infants (69%); 29 (60%) were treated by EVD and 16 (33%) subsequently received a permanent shunt.

Population baseline and follow-up characteristics were stratified by primary endpoint with *p* values for the ‘better outcome group’ (scores above the median) vs the ‘poor outcome group’ (scores less than or equal to the median or death) (Table 1).

Serum NfL levels decreased markedly from 251 pg/mL [median (interquartile range {IQR} 139–379)] at the initial PIVH visit to 15.7 pg/mL [median (IQR 11.1–33.5)] at the final TEA visit, *p* < 0.001. The NfL CSF/serum ratio increased from 44 [median (IQR 26–136)] at the EVD visit to 162 (median [IQR 103–292]) at the EVD-plus visit, *p* < 0.01 (Table 2).

**Table 2:** All NfL measurements per visit

Visits	Days of life median [IQR]	NfL serum pg/mL median [IQR]	NfL CSF pg/mL median [IQR]
PIVH	3 [2, 5]	251 [139, 379]	–
PIVH-plus	6 [5, 8]	330 [215, 463]	–
EVD	16 [12, 19]	236 [74, 532]	10,502 [3309, 53522]
EVD-plus	20 [17, 24]	121 [87, 304]	19,655 [4343, 114877]
CSF 3	23 [20, 27]	–	24,608 [13903, 95879]
CSF 4	32 [29, 35]	–	13,771 [6720, 30798]
TEA	88 [70, 98]	16 [11, 34]	–

*PIVH* peri/intraventricular hemorrhage, *EVD* extraventricular drainage, *CSF* cerebrospinal fluid, *TEA* term-equivalent age

**Fig. 2:**
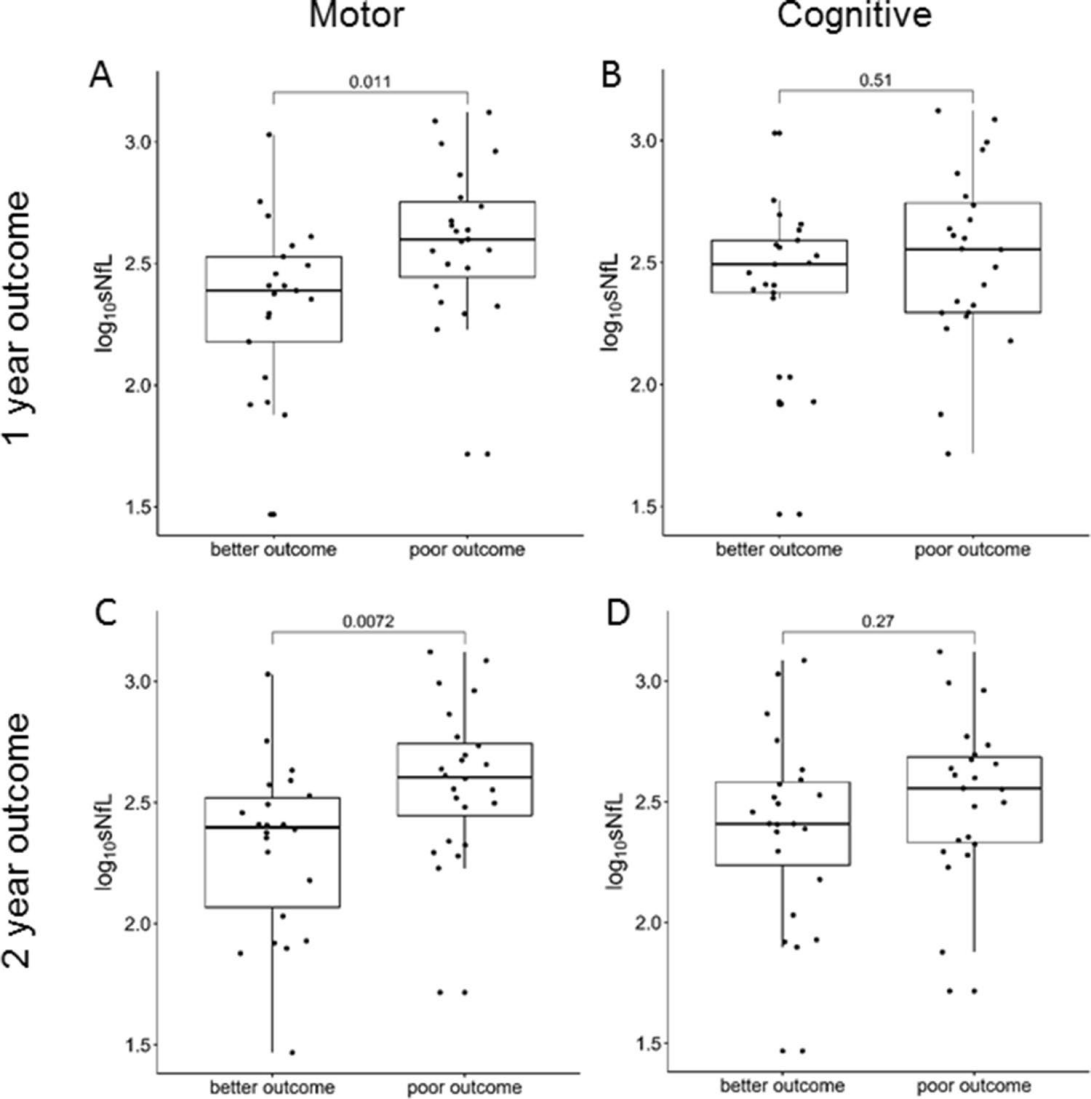
Comparison of serum NfL levels (sNfL) for all composite endpoints; **a** 1-year motor skills, **b** 1-year cognitive skills, **c** 2-year motor skills, and **d** 2-year cognitive skills; *p* values for group differences are from the Wilcoxon-Mann–Whitney test

Infants with poor motor outcome or death at 1 year (*n* = 23) or 2 years (*n* = 24) had significantly higher sNfL levels than those with better motor outcome at 1 year (*n* = 21) or 2 years (*n* = 22) but no differences were seen for cognitive outcome at 1 or 2 years (Table 1, Fig. 2 and Supplemental information). This holds true for the primary endpoint at 2 years when using the upper quartile of the respective scores as cut off instead of the median (Supplemental information).

**Fig. 3:**
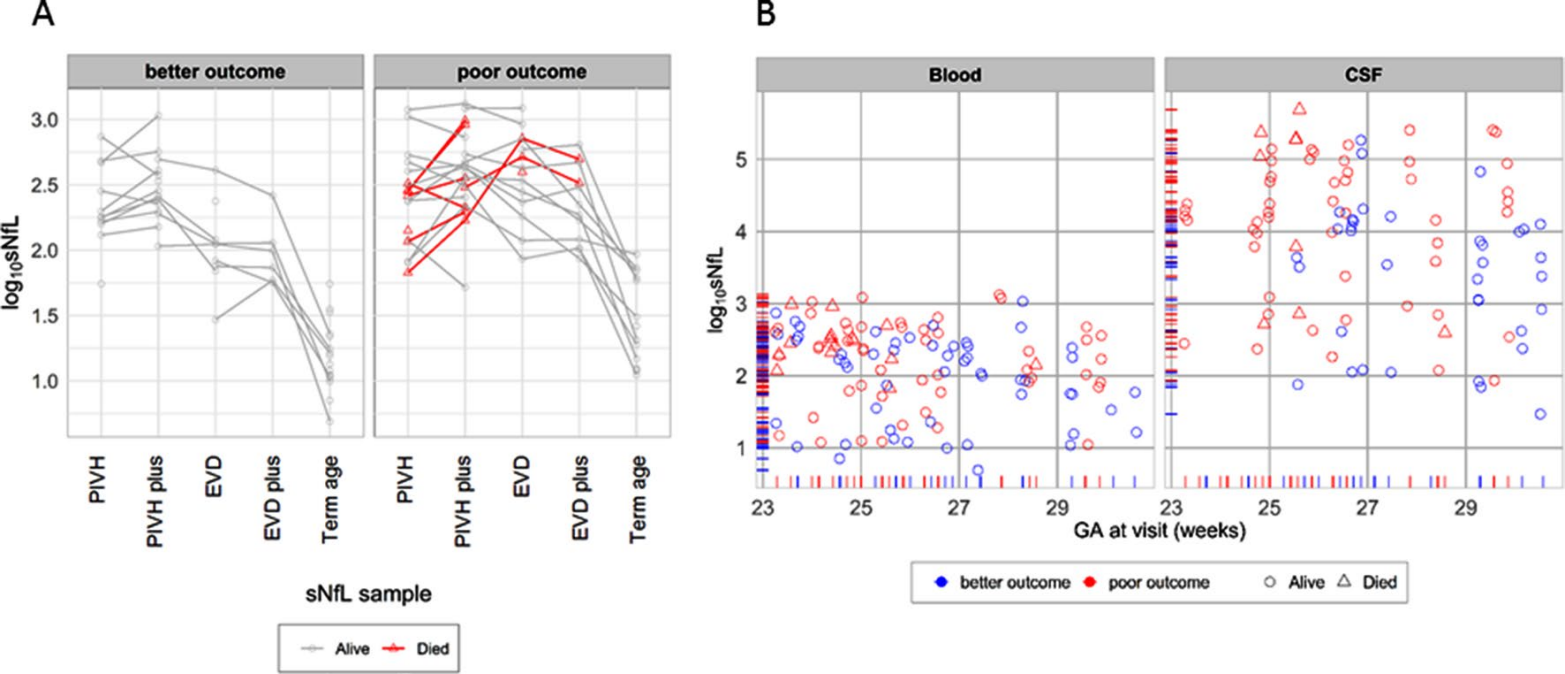
**a** Serum NfL (sNfL) levels at each measurement point stratified by 2-year endpoint into better motor outcome or poor motor outcome or death. **b** NfL levels in serum (left) and CSF (right); those with 2-year poor motor skills (or death) marked in red; deaths marked as triangles; censoring rug plot also stratified by the motor skills or death endpoint

To further illustrate the differences in motor outcome we plotted sNfL trajectories from first to last visit (Fig. 3a) and all NfL values (except serum and CSF at the TEA visit) in each infant in relation to gestational age (Fig. 3b).

**Fig. 4:**
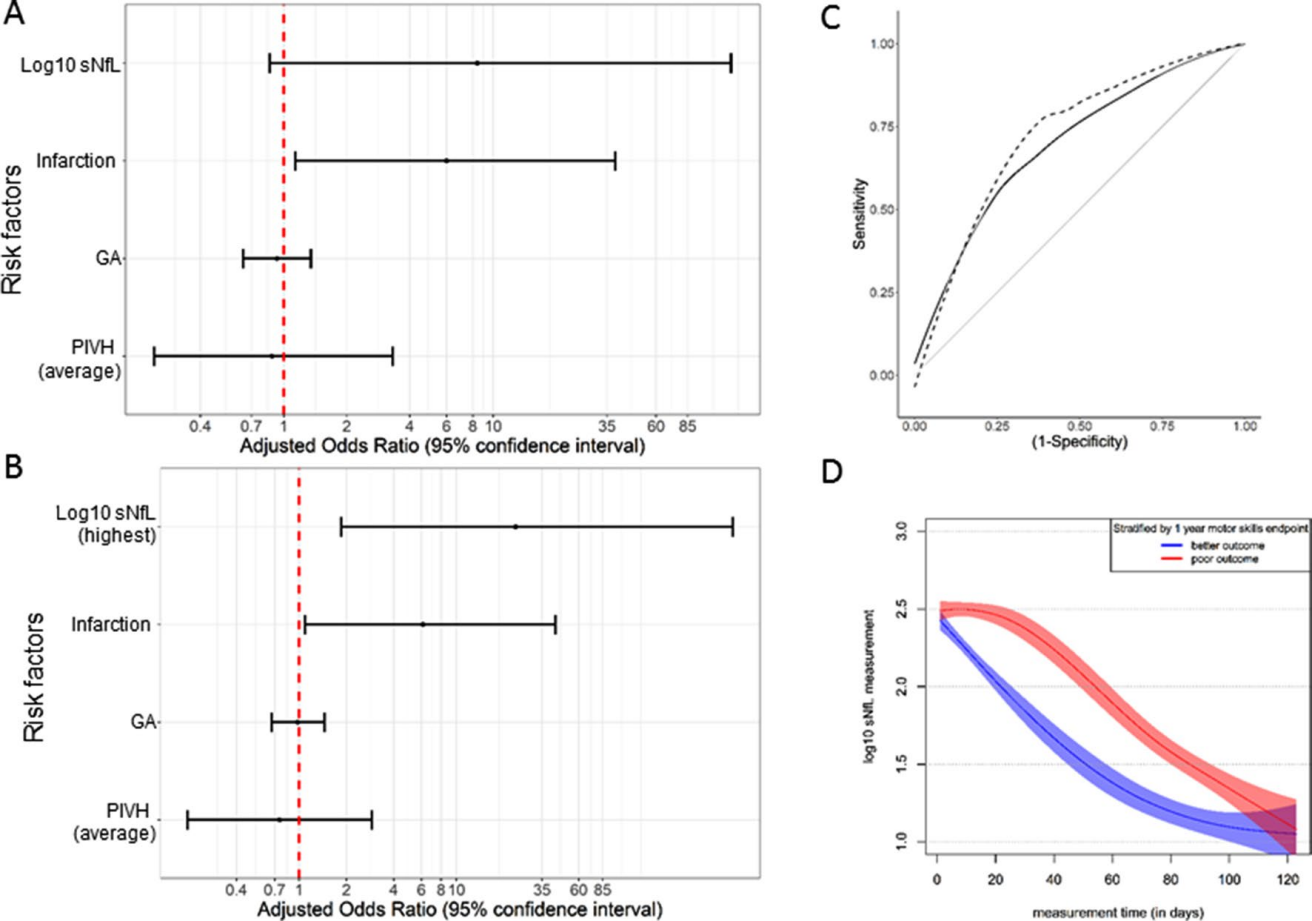
**a, b** Risk factors for the 2-year motor skills (including death) endpoint from the fitted multivariate logistic regression model; variables included based on the univariate analysis (those with *p* value < 0.1). **a** using first log_10_ sNfL measured, PIVH visit; **b** using highest log_10_ sNfL measurement. *GA*, gestational age; *PIVH*, peri/intraventricular hemorrhage. **c** Smoothed receiver operating characteristic (ROC) curves for the predictive value of early serum NfL (sNfL, solid black line) for the 2-year motor skills (or death) endpoint [AUC 0.64 (0.47–0.81)]; also shown is the predictive value of the highest sNfL level [dotted black line, AUC 0.71 (0.54–0.88)]; the nearer the curve is to the top left corner, the better the predictive value. **d** Log_10_ serum NfL (sNfL) measurements plotted against time of measurement (in days) from the general additive model using restricted cubic splines fitted for time with 3 knot points; marginal model stratified by the composite 1-year poor motor skills (or death) endpoint

Next we fitted a univariate and multivariate logistic regression model on the neonatal and maternal characteristics listed in Table 1, with the neurodevelopmental endpoint ‘motor skills at 2 years of age’ as the dependent variable. When including log_10_ sNfL values from the PIVH visit (first sNfL measurement in each patient) in the model, only infarction was an independent risk factor for poor motor outcome or death at 2 years (Fig. 4a). When including the highest log_10_ sNfL values measured, then infarction and highest log_10_ sNfL were independent risk factors for poor motor outcome or death at 2 years (Fig. 4b). ROC curve analysis revealed that highest measured sNfL [AUC 0.71, 95% confidence interval (CI) 0.54–0.88] outperformed PIVH-visit sNfL (AUC 0.64, CI 0.47–0.81) in predicting poor motor skills or death at 2 years (Fig. 4c). sNfL trajectories including all values in all patients also differed clearly between the two groups (Fig. 4d).

In the mixed-effects linear model with sNfL as the dependent variable, time of sNfL measurement (*p* < 0.001), poor motor outcome or death at 2 years (*p* = 0.015), and gestational age at birth (*p* = 0.004) had significant independent influence on sNfL, whereas infarction, highest grade PIVH, and death were significant in the univariate but not in the mixed-effects linear model, very similar findings were noted for the 1-year endpoint (Table 3).

**Table 3:** Factors affecting sNfL levels

Variable	Univariate		Multivariate	
	Estimate (95% CI)	*p*	Estimate (95% CI)	*p*
Time of measurement (days)	− 0.014 (− 0.015, − 0.012)	** < 0.001**	− 0.014 (− 0.014, − 0.012)	** < 0.001**
Poor motor outcome or death at 2 years	0.34 (0.16, 0.53)	**0.001**	0.28 (0.11, 0.45)	**0.004**
Infarction	0.22 (0.04, 0.40)	**0.02**	− 0.08 (− 0.28, 0.12)	0.44
Gestational age (weeks)	− 0.06 (− 0.10, − 0.01)	**0.02**	− 0.06 (− 0.10, − 0.02)	**0.007**
PIVH grade (average)	0.20 (0.06, 0.34)	**0.008**	0.11 (− 0.04, 0.26)	0.16
Died	0.37 (0.10, 0.65)	**0.009**	− 0.23 (− 0.48, 0.03)	0.09

Estimates from fitting univariate models (left) with dependent variable log_10_ sNfL level at time (in days) of the measurements (IVH_blood, IVH-plus_blood, EVD_blood, EVD-plus_blood, term_blood); estimates from fitting the fully adjusted multivariate model (right) with random intercept and slope effects for the trajectory of each patient over time (48 patients, 153 measurements); variables with a significant effect in univariate models (*p* < 0.1) were included in the multivariate model. Included are all patients and all measurements. Non significant at the 5% level in univariate models: sex, post-hemorrhagic hydrocephalus, *AIS* amnion infection syndrome, neonatal infection, shunt infection, Cesarean section, antenatal steroids, postnatal steroids, mother’s age, *SES* socio-economic standard, Apgar; birth weight had a significant effect (*p* = 0.03), but was colinear with gestational age and, therefore, not included in the analysis. First-level interaction between time and primary endpoint was non significant at the 5% level

## Discussion

Our study investigated the role of neuroaxonal damage protein NfL in serum and CSF in predicting neurodevelopmental outcome in preterm infants with severe brain damage, namely intraventricular hemorrhage or periventricular infarction, in the first days of life. Our main findings were that (1) sNfL levels depend on maturity, birth weight, postnatal age at measurement and brain damage severity, (2) sNfL levels are higher in infants with the composite outcome of poor motor skills or death at 1 and 2 years of age, and (3) sNfL is an independent predictor of motor but not cognitive outcome.

A considerable strength of the study is the availability of serial sNfL measures from initial PIVH diagnosis in the first days of life through term equivalent age (TEA), enabling the investigation of within-person sNfL dynamics over many weeks. Average sNfL levels were 20-fold higher at PIVH diagnosis than at TEA 12 weeks later (Table 2). sNfL peaked several days after PIVH diagnosis in most infants, with individual dynamics most likely reflecting the extent of ongoing neuroaxonal damage. Outcome was significantly poorer in infants with persistently high sNfL levels than in those whose levels fell sharply (Fig. 4d). Thus it is the dynamics of sNfL in an individual rather than the absolute level that is clinically predictive, as has recently been shown, even years in advance of symptom onset, in Alzheimer’s disease and amyotrophic lateral sclerosis [8].

An additional strength of our study is the uniform treatment received by all infants, with particular regard to the management of PHH. CSF was removed whenever PHH exceeded an a priori defined degree [10]. In general, over the long term, the infants most affected in terms of neurodevelopmental outcome are those with severe PIVH, especially those with secondary PHH [14]. Recent evidence from an observational cohort study favors early PHH relief [15] but a multicenter randomized controlled trial failed to identify the optimal timing for intervention [16]. The uniform treatment in our study explains why neither PHH per se nor subsequent complications such as ongoing PHH, with the necessity for a permanent shunt or shunt infections, were identified as outcome predictors.

Severe (grade 3–4) PIVH carries a greater risk of moderate to severe neurodevelopmental impairment or death than mild to moderate or no PIVH [2]. In our study confined to infants with moderate to severe PIVH, the severity of PIVH and particularly the presence or absence of infarction were associated with neurodevelopmental outcome at both 1 and 2 years of age. We identified sNfL as an independent predictor of motor but not cognitive outcome (Figs. 2, 4). There might be several reasons why sNfL is associated with motor but not cognitive development. First, susceptibility of brain regions and their neurons for motor and cognitive systems differs [5]. Second motor and cognitive performance might be captured with varying sensitivity and specificity in the Bayley-III test battery [11], thus, our study might be underpowered for the cognitive outcome. Third, our data indicate an increasing trend from 1 to 2 years of age towards an association of poor cognitive outcome and higher sNfL (Fig. 2), indicating that in a larger cohort sNfL might be associated not only with motor but also with cognitive outcome.

The sNfL levels we found in infants with moderate to severe PIVH (median 251 pg/ml) exceed those reported for infants with mild to moderate PIVH (median 212 pg/mL) or no brain damage (median 123 pg/mL) at the same postnatal age of about 1 week [9]. They also exceed levels in adults with various neurological conditions [12,17], stroke [18] or brain trauma [19,20]. Direct comparison is not possible due to assay differences. Adults with HIV-associated dementia, which affects the brain extensively, show similarly high levels [21].

By the TEA visit, sNfL levels had decreased to 15.7 pg/mL, approximating to those reported for at term born healthy infants (18.2 pg/mL) [9] and only slightly higher than those in healthy children and adults (10 pg/mL) [12,22]. High levels in the first days of life may reflect the heavy neuroaxonal damage caused by severe PIVH, as well as high neuron turnover and vulnerability in the postnatal phase. Our multivariate linear regression analyses with sNfL as the dependent variable identified infarction, average grade of PIVH, gestational age and birth weight as the significant predictors of sNfL. However, serum levels peaked at the end of the first week of life (median 330 pg/mL), whereas CSF levels did not peak until the fourth week of life (median 24,608 pg/mL). These staggered dynamics might be explained by the maturation of various blood–brain barrier components during gestation and adaptation to extrauterine life and spatio-temporal differences in hypoxia and blood circulation ^23,24^.

In summary, we have shown that sNfL dynamics in preterm neonates with PIVH can predict motor outcome at 1 and 2 years of age. We need further studies to assess sNfL as a predictor of neurodevelopmental outcome in the preterm population in general. Techniques such as amplitude-integrated electroencephalography and magnetic resonance imaging could reveal correlations within neurophysiology and neuroimaging. Meanwhile, our findings may help to pave the way for sNfL as a clinical biomarker in brain-damaged preterm infants.

## Supplementary Information

Below is the link to the electronic supplementary material.

Supplementary file1 (PPTX 78 KB)

## Data Availability

Anonymized data not published within this article will be made available by request from any qualified investigator.
