# Supplementary material for: Early life serum neurofilament dynamics predict neurodevelopmental outcome of preterm infants
Source: J Neurol. 2021 Feb 10;268(7):2570–7. doi: 10.1007/s00415-021-10429-5 (PMC8217001; doi:10.1007/s00415-021-10429-5)
Supplement: Supplementary file 1 — Supplementary file1 (PPTX 78 KB) [file 415_2021_10429_MOESM1_ESM.pptx]

## Slide 1
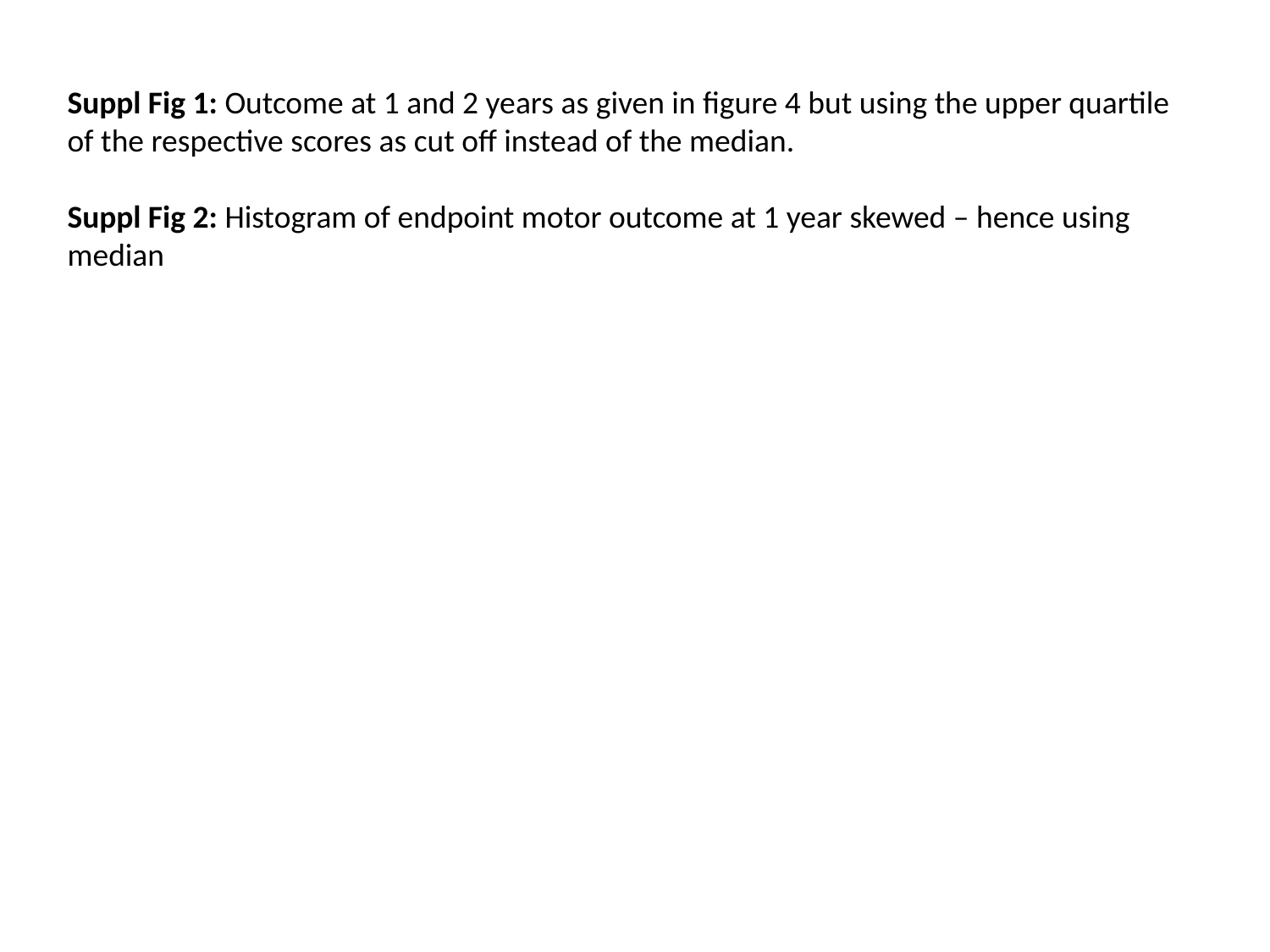

Suppl Fig 1: Outcome at 1 and 2 years as given in figure 4 but using the upper quartile of the respective scores as cut off instead of the median.
Suppl Fig 2: Histogram of endpoint motor outcome at 1 year skewed – hence using median
#

## Slide 2
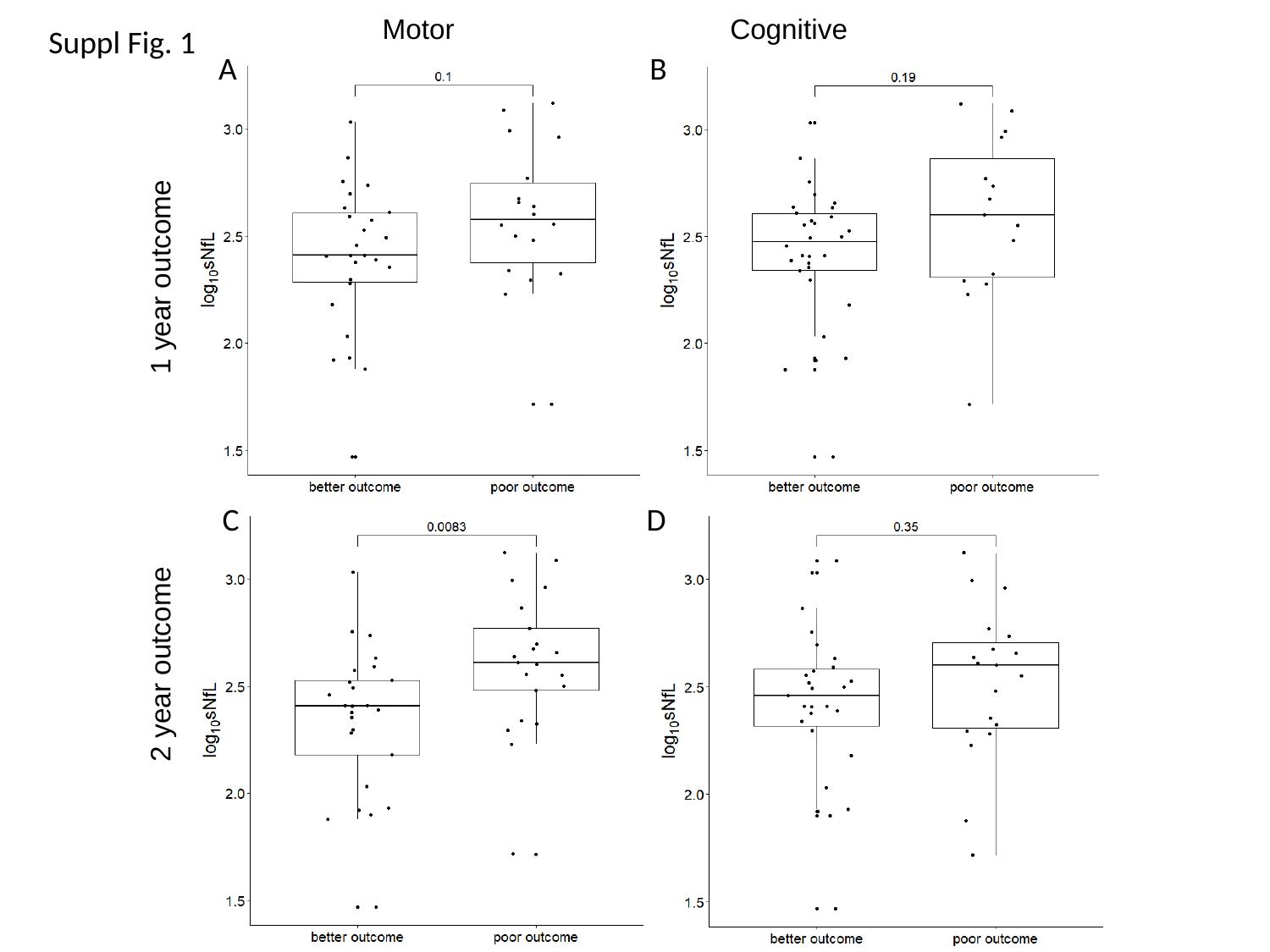

Motor 		 Cognitive
Suppl Fig. 1
A			 B
2 year outcome 	 1 year outcome
C			 D

## Slide 3
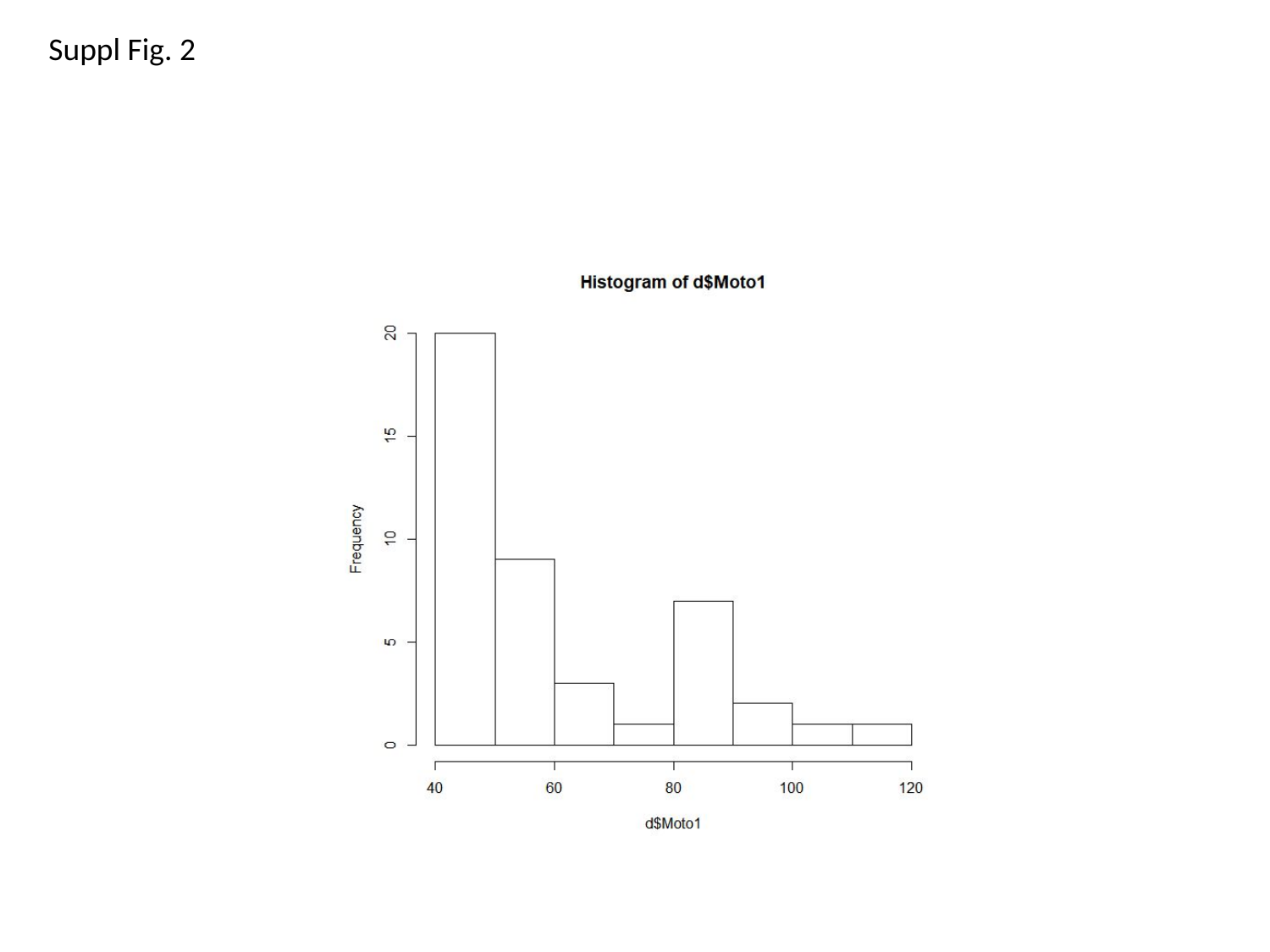

Suppl Fig. 2
